# Hitting Close to Home: The Effect of COVID-19 Illness in the Social Environment on Psychological Burden in Older Adults

**DOI:** 10.3389/fpsyg.2021.737787

**Published:** 2021-09-27

**Authors:** Lukas Richter, Theresa Heidinger

**Affiliations:** ^1^Department of Social Sciences, St. Pölten University of Applied Sciences, St. Pölten, Austria; ^2^Department of General Health Studies, Division Gerontology and Health Research, Karl Landsteiner University of Health Sciences, Krems, Austria

**Keywords:** social environment, psychological burden, COVID-19, depression and anxiety, secondary traumatic stress, effects of the pandemic on mental health, older adults

## Abstract

This study examines the impact of COVID-19 experience of infection in the individual’s social environment on psychological burden controlling for a broad range of factors using data on an older population (50+ years). Based on the empirical evidence of preexisting studies, it is hypothesized that psychological burden will increase concurrent to the severity of COVID-19 experience (tested positive, hospitalized, and death) independent of the other stressors resulting from the pandemic, such as a subjective sense of uncertainty or financial burden. Data of the Survey of Health, Aging and Retirement in EUROPE, and a European cross-national panel study were used to examine this hypothesis. Besides Chi^2^ test and Spearman’s rho, a logistic regression model was constructed to test the hypothesized model. The study confirms that there is significantly higher risk for psychological burden by heightened COVID-19 severity in the social environment independent of multiple also significantly influential variables depicting stressors to everyday life of older people during the pandemic. The results point to the importance of multiple factors (social, financial, health, and sociodemographic) which have significantly affected the psychological condition of the individual during the past year. Conclusively, the results illustrate the dilemma that infection and illness in the social circle, as well as countermeasures (social distancing), have negative consequences for our mental health.

## Introduction

The global pandemic and the ensuing safety measures have had a major (mostly unfavorable) impact on the everyday lives of large parts of the population. From a gerontologists perspective, this is especially true for older people, whose life has been subjected to multiple burdens over the past year. This group has been, and continuous to be, considered at risk for severe illness and mortality from COVID-19 infection (Shahid et al., 2020; Gerwen et al., 2021) and therefore has had to incorporate changes into their day to day life. Health policies have targeted older people in asking them to isolate and physically distance to avoid infection which, although successful in protecting from the virus, have had negative consequences of their own: Increased subjective social isolation (Peng and Roth, 2021), lower healthcare utilization (Ksinan Jiskrova et al., 2021), reduction of physical activities (Creese et al., 2020; Brown et al., 2021), and social interaction (Richter and Heidinger, 2020; Heid et al., 2021) as well as the ever increasing problem of loneliness (Carson et al., 2020; Entringer et al., 2020; Heidinger and Richter, 2020; Luchetti et al., 2020; Stolz et al., 2020; Krendl and Perry, 2021) have been reported as byproducts of COVID-19 safety measures. As these lifestyle changes have been previously linked to adverse mental health outcomes, it is not surprising that an increase in depression, anxiety and stress symptoms, and a general decline of mental health during the pandemic (Shahid et al., 2020; Gerwen et al., 2021) have been noted in multiple studies (Banks and Xu, 2020; Bailey et al., 2021; De Pue et al., 2021; Grolli et al., 2021; Tsoukalis-Chaikalis et al., 2021). These issues seem to be greater in younger population cohorts (Banks and Xu, 2020; Carson et al., 2020) but have been found to be universal among (western) populations.

### Unfavorable Consequences of the Pandemic on the Mental Health of Older People

Practices, such as withdrawal (Skoog, 2020) and cocooning (Bailey et al., 2021) with the accompanying decrease in social contact and increase in feelings of loneliness (Okruszek et al., 2020; Clair et al., 2021), have been identified as influential for increased psychological burden among older people. In addition, experience of COVID-19-related ageism (Skoog, 2020), perception of increased risk of COVID-19 illness (Sigurvinsdottir et al., 2020), fear due to the virus (Warren et al., 2021), experience of COVID-19 infection (Sigurvinsdottir et al., 2020; Silver, 2020), and decrease in physical activity (Creese et al., 2020) have been found to adversely impact mental health. This impact is moderated by the psychological makeup of individual encompassing personality traits (Wei, 2020), emotion regulation ability (Prout et al., 2020), coping behaviors (Minahan et al., 2021), anxiety sensitivity (Warren et al., 2021), and social resources (Litwin and Levinsky, 2021).

### Lessons From SARS and Research Goal

Even though the literature on the psychological effect of the pandemic is growing, it is still worth sharing Gallagher’s et al. (2020) sentiment that little empirical work has addressed how differential COVID-19 experiences of infection, illness, and death in the social environment affect the mental health of individuals. Experience of disease is an important factor for mental health, which had been previously shown in studies pertaining to the SARS outbreak in the early 2000s. Hawryluck et al. (2004) noted that having persons in one’s social circle hospitalized with SARS increased the probability of exhibiting symptoms of posttraumatic stress and depression (Hawryluck et al., 2004). This finding was mirrored in a later study focusing on healthcare workers, where having an infected friend or family member increased symptoms of posttraumatic stress disorder (PTSD) by a factor of three (Wu et al., 2009). Utilizing these findings as starting point, it is interesting to ask whether similar findings have been reported during the present, far longer-lasting, pandemic. An overview of relevant studies published up until this point in time is presented below.

### Current State of Research on Mental Health Burden During COVID-19

In a study surveying the Spanish population at an early stage of the pandemic (March of 2020), researchers showed that having a close relative infected with the disease was positively related to anxiety, depression, and PTSD (González-Sanguino et al., 2020). A comparable finding was reported by Mazza et al. (2020) who surveyed the general population in Italy in the first moths of the pandemic: Psychological burden was increased among people who had experienced COVID-19 illness in their personal network. They reported increased anxiety to be a consequence of a family member being infected with the virus, while increased depression followed the infection of acquaintances (Mazza et al., 2020). In a similar vein, an Australian team reported depression, anxiety, and stress as well as PTSD symptoms to be significantly higher in persons who had themselves come into contact with the disease or had someone in their social circle infected with, hospitalized with, or pass away from the virus (Bridgland et al., 2021). Anticipated negative outcomes of the pandemic, of which the most commonly mentioned and most feared was that a family member or close friend falls ill and dying from the virus, were also shown to affect PTSD, depression, anxiety, and stress measures which indicates that worry about the welfare of the social circle may itself have a powerful impact on mental health. Similarly, an American study demonstrated that COVID-19 illness in the social environment strongly influences participants subjective stress levels, finding that worry for others had a measurable impact on the individuals’ mental health: Authors reported that the “risk of loved ones becoming infected” had the second highest stress rating after the “loss of job security and income” and was mentioned by 61% of the respondents (Park et al., 2020). This finding is in accordance with Gallagher et al. (2020) who reported significant relationships between anxiety and depression and COVID-19 experience, such as “having a COVID-19 related death in one’s proximity” or “knowing someone who was infected with the virus.” They were able to show that the latter tripled the chance of receiving a depression or anxiety diagnosis (comparable with Cao et al., 2020), while the former increased probability of depression diagnosis by a factor of 5 and anxiety diagnosis by a factor of 6.

These findings have provided indications that lived experience of COVID-19 illness of “other” (as opposed to own) affects mental health differentially, but generally aversely. This can be interpreted in the context of secondary traumatic stress defined as “the natural, consequent behaviors and emotions resulting from knowledge about a traumatizing event experienced by a significant other. It is this stress resulting from helping or wanting to help a traumatized or suffering person” (Figley, 1999, S. 10). The symptoms of secondary trauma are close to PTSD symptoms with the “by standing” person developing adverse psychological reactions to the experience of another, most commonly close, individual. These reactions can include stress and anxiety to depression and somatization among more severe PTSD symptoms, providing a comprehensive picture of life during the pandemic and its impact on mental health.

### Résumé and Research Question

Thus far, a more in-depth analysis of psychological burden by COVID-19 experience on the foreground of a larger, holistic view of the individuals lived experience during the pandemic is lacking. While Gallagher et al. (2020) distinguished between other-illness severity levels, they did not include any other potentially influential variables concerning the lives of the respondents. Mazza et al. (2020) and González-Sanguino et al. (2020) conducted multivariate analyses without differentiating the severity of COVID-19 experience thereby leaving a gap in the research. Finally, these studies have been conducted among the general population, while, to the best of our knowledge, none to date have analyzed the specific effect on the older population.

This study will provide an examination of the impact of COVID-19 experience of infection in the individual’s social environment staggered by severity on psychological burden controlling for a broad range of factors using data on an older population (50+ years). Burden, as used in this study, encompasses multiple adverse psychological states (self-reported feelings of anxiety, depression, and troubled sleep) which conjointly result in an unfavorable mental condition. Based on the empirical evidence of the preexisting studies, it is hypothesized that psychological burden will increase concurrent to the severity of COVID-19 experience independent of the other included factors.

## Materials and Methods

### Sample Design

Data of the Survey of Health, Aging and Retirement in EUROPE (SHARE), and a European cross-national panel study were used to examine this hypothesis.^1^ To this end, wave 8 and wave 7 data were combined to construct a more comprehensive model of the respondent’s life situation. The wave 8 – COVID-19 Survey 1 – release version: 0.01 (Börsch-Supan, 2020b) was conducted from June to July 2020 in 27 countries and focused specifically on life experience during the global pandemic. Data from wave 7 release version: 7.1.1 (Börsch-Supan, 2020a) collected in 2017 were used to impute information on personality traits and supplementary demographic information of the respondents.

Nine Percent (i.e., 4,715 persons) of the 52,310 participants of the wave 8 survey indicated that they knew someone who had been (1) tested positive, (2) hospitalized, or (3) had passed away due to a COVID-19 infection. As data collection was conducted in the summer months of 2020 during the first wave of the pandemic, which, compared to later waves in autumn and winter of 2020, was relatively small in large parts of Europe with heterogeneous prevalence rates between European countries, this study only included data from countries, where at minimum 5% of respondents had indicated COVID-19 experience as described above. Sorted by the prevalence rate among respondents provided here in brackets these countries are: Luxembourg (24.5%), Belgium (24.1%), Sweden (21.4%), Netherlands (19.6%), Switzerland (18.3%), Spain (18.2%), Denmark (15.1%), Portugal (14.3%), France (13.9%), Italy (13.3%), Israel (10.3%), Germany (8.8%), and Malta (7.5%). Additionally, respondents (*n*=273 or 0.5% of the whole sample) who indicated having had a COVID-19 infection themselves were filtered out in order to exclude any spillover effect of own infection experience. Furthermore, data gathered by proxy interviews were filtered as well. The resulting sample included 22,776 participants and was made up of 57% female participants. Mean age was 70.73years (*SD*=8.83years, range 50–104years) with 25% of the sample living alone during the pandemic. Education was distributed as follows: 37% no education – ISCED 2, 35% ISCED 3–4, and 28% ISCED 5–6.

### Measures

An overview of all measures is provided in Table 1. To construct the dependent variable of psychological burden, data of six individual items were combined. First, persons were asked whether they had felt (1) nervous, anxious or on edge, (2) sad or depressed or whether they had (3) trouble sleeping which they could affirm or deny. In case of an affirmative answer, a follow-up question of change in frequency as compared to life before the pandemic was asked (“Has that been more so, less so, or about the same as before the outbreak of Corona?”). Descriptive information on these items is provided in the first part of the results section. As burden is a comprehensive concept informing on adverse mental health states, authors did expect moderate intercorrelations among the individual psychological measures (correlations among measures of depression, anxiety, and disturbed sleep ranged from Phi=0.31 to 0.49). Aside from prior knowledge on the relationship between these psychological states (Staner, 2003; Nutt et al., 2008; Tiller, 2012), these moderate to strong intervariable relationships provide a statistical basis for the construction of this variable. For multivariate analysis, items were combined and dichotomized as: (0) no reported psychological burden or similar or lesser burden as compared to before the pandemic and (1) reported higher psychological burden in one or more adverse psychological states.

**Table 1:** Operationalization.

Variable	Manifestation	
Psychological burden	0 ‘no reported psychological burden or similar or lesser burden as compared to before the pandemic” 1 “reported higher psychological burden in one or more adverse psychological states (nervous, anxious or on edge, and/ or sad or depressed, and/or trouble sleeping)”	
Severity of COVID-19 experience	0 “No COVID in social environment” 1 “Anyone tested positive for COVID-19” 2 “Anyone hospitalized due to COVID-19” 3 “Anyone died due to COVID-19”	
Health vulnerability	subjective health status prior to the pandemic	1 “Excellent/very good” 2 “Good” 3 “Fair/poor”
	Health status during the pandemic	0 “Same or improved” 1 “Deteriorated”
	Neuroticism	1 (low) – 5 (high) scale
Variables describing subjective loss of control	Postpone medical appointment	0 “no” 1 “yes”
	Denied medical appointment	
	Receive help to obtain necessities since outbreak	
	Able to make ends meet	1 “with great difficulty” 2 “with some difficulty” 3 “fairly easily” 4 “easily”
Sociodemographic variables	Highest formal education	1 low “(= ISCED 97; 0,1 and 2)” 2 middle “(= ISCED 97; 3 and 4)” 3 high “(= ISCED 97; 5 and 6)”
	Household size	Metric
	Age	
	Gender	1 “male” 2 “female”

COVID-19 experience was defined as subjective encounter with the virus in the individuals social circle,^2^ whereby experiences were categorized by their severity: (0) no COVID-19 case, (1) positive COVID-19 test, (2) hospitalization, and (3) mortality due to the virus. The resulting variable was termed severity of COVID-19 experience (SoCE) and focused solely on the witnessed experiences of other, close persons in the individuals social circle. When multiple experiences were reported by the respondent, the most severe experience was used on this variable.

Multiple control variables were introduced to test the hypothesized association between psychological burden and SoCE in the context of the unusual circumstances due to the pandemic. These had been previously linked to increased psychological burden or, respectively, had presented as stressors during the pandemic and can be summed up into three dimensions: health vulnerability, variables describing subjective loss of control, and sociodemographic factors.

Health vulnerability was depicted using three variables: (A) subjective health status prior to the pandemic which was coded on a scale of (1) excellent/very good, (2) good, and (3) fair/ poor; (B) perceived change in health status since the outbreak of the pandemic coded as (0) same or improved and (1) deteriorated, and (C) psychological susceptibility to adverse effects of the pandemic proxied by neuroticism. Persons who score high on this personality trait are generally more prone to uncertainty, nervousness, anxiety, and depression (Lahey, 2009). Caci et al. (2020) suggested that neuroticism is influential for coping with the COVID-19 pandemic, as highly neurotic people show greater emotional reactivity and exhibit fewer resources for stress management. They also reported a positive correlation between neuroticism and COVID-19 health anxiety (also Lee et al., 2020; Nikčević et al., 2021). Therefore, the neuroticism score extracted from the Big Five Inventory (BFI-10, Rammstedt et al., 2013) was added into the computed model. The score ranges from 1 to 5 with higher scores depicting higher neuroticism.

It has been previously shown that an internal locus of control is beneficial to mental health (Cheng et al., 2013), with a study conducted during the pandemic finding an association between external locus of control and adverse mental health states, such as anxiety, depression, and stress (Sigurvinsdottir et al., 2020). This led us to include variables describing a subjective loss of control which may separately contribute to psychological burden. The first variables concern the suspension of medical care using the following questions: “Did you have a medical appointment scheduled, which the doctor or medical facility decided to postpone due to Corona?” and “Did you ask for an appointment for a medical treatment since the outbreak of Corona and did not get one?” (each: 1=yes and 0=no). Additionally, a measure of social dependence was added into the model: “Since the outbreak of Corona, were you helped by others from outside of home to obtain necessities, e.g., food, medications or emergency household repairs?” (1=yes and 0=or no). Finally, participants were asked to provide information on their financial situation during the pandemic: “Would you say that your household is able to make ends meet with great difficulty, with some difficulty, fairly easily, or easily?”, which was scored on a scale (1) great difficulty to (4) easily.

Sociodemographic variables were also added into the model. Level of formal education rated using the ISCED 1997 system was included as education had been previously associated with psychosocial resources (Niemeyer et al., 2019). Education was summarized into three categories (1) low [ISCED 0–2], (2) middle [ISCED (3–4)], and (3) high [ISCED (5–6)]. Additionally, household size, measured as a metric variable, was included, as larger households had previously been shown to protect against the negative impacts of the pandemic (Groarke et al., 2020). Finally, the model also introduces age as a metric variable and gender as nominal variable (1) male and (2) female.

### Statistical Analysis

Data analysis was conducted using IBM SPSS 26. Unweighted data were used for analyses. For bivariate analyses of the association between SoCE and the variables of burden Chi^2^ test with *Bonferroni* adjusted *post-hoc* tests and Spearman’s rho were computed. In order to test the hypothesized influence of SoCE on psychological burden over and above all other variables, a logistic regression model was constructed using psychological burden as the dependent variable and introducing SoCE as well as all mentioned control variables as explanatory variables.

## Results

Table 2 provides an overview of the sample distribution on the captured adverse psychological states (feeling nervous/anxious/on edge, feeling sad/depressed, and having trouble sleeping) as well as bivariate analysis. 23.9% of participants felt they had become more anxious or nervous during the pandemic, 19% reported feeling more depressed, and 9% had more trouble sleeping than before the pandemic.^3^ Analyzing the impact of SoCE among individuals who indicated an increase in psychological stressors revealed a relationship between these variables: The more severe the degree of infection in the social environment, the more likely respondents reported an increase in adverse psychological states, particularly when someone in the social environment had passed away due to the virus (significant differences between no/tested positive/hospitalization vs. death due to infection among all three adverse states). However, Spearman’s rho, are small, illustrating weak correlations.

**Table 2:** Univariate and bivariate analysis of psychological burden and COVID-19 cases.

Adverse psychological state	No	Yes			N
		Less so	About the same	More so	
nervous/anxious/ or on edge	69.3%	0.5%	6.3%	23.9%	22,605
sad/depressed	73.6%	0.5%	6.9%	19.0%	22,587
trouble sleeping	75.2%	0.5%	15.3%	9.0%	22,609
					
**State Change (more so)**	**COVID-19 cases in the social environment**				**Spearman’s rho**
	**No**	**Tested positive**	**Hospitalized**	**Died**	
Nervous/anxious/ or on edge (more)	23.1%_a_	25.7%_a_	26.8%_a,b_	31.6%_b_	0.043; *p<* 0.01
Sad/depressed (more)	18.4%_a_	18.9%_a_	20.6%_a_	26.7%_b_	0.035; *p<* 0.01
Trouble sleeping (more)	8.7%_a_	8.9%_a_	9.9%_a_	13.4%_b_	0.027; *p<* 0.01

Subscript letters represent the *Bonferroni* adjusted post-hoc tests: Values with a significantly differ from values with b.

The analyzed sample in the logistic model includes 18,586 observations due to missing values. With Nagelkerke’s *R*^2^ at 0.158, the Hosmer-Lemeshow test at 0.078 and ROC AUC at 0.703, the model is deemed acceptable. Odds ratios are presented in Table 3. Within the model, SoCE is shown to be a predictor of increased psychological burden over and above all other variables. The association follows the hypothesized direction as the likelihood for increased psychological burden surges with increasing SoCE: tested positive (*OR*=1.257; CI 1.107–1.427); hospitalization (*OR*=1.330; CI 1.119–1.582); and death due to the virus (*OR*=1.579; CI 1.371–1.819). It can be concluded that the more serious the COVID-19 experiences in the persons social environment, the higher the risk of increased burden – however, as the CIs of the point estimators overlap, this increase of the ORs should be interpreted with caution.

**Table 3:** Logistic regression model predicting psychological burden.

		Odds Ratio	95% CI		Wald	*P*
Severity of COVID-Experience (SoCE)	COVID in the social environment (ref. no COVID-19 cases)					
	Anyone tested positive	1.257	1.107	1.427	12.461	< 0.01
	Anyone hospitalized due to COVID-19	1.330	1.119	1.582	10.448	< 0.01
	Anyone died due to COVID-19	1.579	1.371	1.819	39.970	< 0.01
Health vulnerability	Subjective health (ref. excellent/ very good)					
	Good	1.314	1.209	1.428	41.179	< 0.01
	fair/poor	1.951	1.770	2.150	181.575	< 0.01
	Health change (ref. improve or same)	3.694	3.296	4.139	506.219	< 0.01
	Neuroticism (Big Five)	1.258	1.218	1.300	188.630	< 0.01
Control-relevant burdens	Postpone medical appointment (ref. no)	1.216	1.133	1.305	29.197	< 0.01
	Denied medical appointment (ref. no)	1.338	1.163	1.539	16.673	< 0.01
	Receive help in obtaining necessities since outbreak (ref. no)	1.267	1.170	1.372	34.092	< 0.01
	Able to make ends meet (ref. easily)					
	Fairly easily	1.084	1.003	1.171	4.119	< 0.05
	With some difficulty	1.395	1.264	1.539	43.955	< 0.01
	With great difficulty	1.676	1.423	1.974	38.188	< 0.01
Control Variables	Highest formal education (ref. low)					
	Middle	0.831	0.767	0.901	20.387	< 0.01
	High	0.938	0.858	1.026	1.939	0.164
	Household Size	1.011	0.970	1.053	0.261	0.610
	Age	0.984	0.980	0.988	51.565	< 0.01
	Gender (ref. women)	0.548	0.511	0.587	289.339	< 0.01
	*X*^2^/df/p	2231.268/18/0.000				
	Nagelkerke’s *R*^2^	0.158				
	*n*	18,586				
	Hosmer-Lemeshow/ ROC AUC	0.078/0.703				

Values in bold are significant (p < 0.05).

As seen in Table 3, most of the included variables significantly impact psychological burden with negative health change having the largest effect (*OR*=3.694; CI 3.296–4.139). Participants subjective health and neuroticism levels both influence the likelihood of psychological burden, as do the variables describing a subjective loss of control. It is also notable that postponed and denied medical appointments as well as new dependencies to obtain necessities since outbreak increase the risk of psychological burden. Having a member of the social circle die of the virus is roughly comparable to experiencing serious financial strain during the pandemic in the likelihood of reporting increased psychological burden (great difficulty to make ends meet during the pandemic, *OR*=1.676; CI 1.423–1.974).

Included sociodemographic variables are partly associated with psychological burden: A moderate level of education and rising age (OR=0.984; CI 0.980–0.988) leads to lower risk of an increased psychological burden during the first months of the pandemic. Women are twice as likely to report psychological burden than men (*OR*=0.548; CI 0,514–0,590) while household size does not significantly predict psychological burden.

## Discussion

This study presents evidence of increased psychological burden among persons 50+ during the pandemic with close to every 10th respondent reporting heightened trouble sleeping, every fifth respondent being more sad or depressed, and close to every fourth person feeling more anxious, on edge, or nervous. These results are striking, considering that data were collected during the first wave of the pandemic which was relatively small compared to the second and third wave experienced over the autumn and winter months. However, as COVID-19 was a novel, largely unknown infectious disease at this time, it may have led to higher anxiety and feelings of distress, which may have leveled off over the following months (Bendau et al., 2021).

Ultimately, the ventured hypothesis of increased psychological burden by COVID-19 severity in the social environment can be confirmed by this study. The effect of SoCE remains significant, if slightly smaller than previously reported (Gallagher et al., 2020; Mazza et al., 2020), after the inclusion of multiple variables depicting stressors to everyday life during the pandemic. This result shows the importance of multiple factors (social, financial, health, and sociodemographic) which have significantly affected the psychological condition of the individual during the past year. Results of this study demonstrate this for older age cohorts. Although older age presents as somewhat of a protective factor against increased psychological burden, which may be explained by psychological resilience (Gooding et al., 2012), COVID-19 experience in the social circle remains a significant contributor to increased psychological burden in persons of 50+ years.

The stressor “risk of loved ones becoming infected” described in Park et al. (2020) is shown to truly affect psychological burden with increased severity of infection experience coinciding with a heightened risk of occurrence of adverse psychological states. These findings can be interpreted as secondary traumatic stress or as consequence from knowledge about a traumatizing event experienced by significant others. Also, COVID-19 experience in the social circle may well have helped substantiate an, at that point rather abstract, illness contributing to the feeling of threat to the environment but also to own health. As Berger and Luckmann, 1990 ascertained, reality is shaped by the experiences of the body in the present time; therefore, the actuality of the threat of COVID-19 illness in a person’s social circle may well have changed the assessment of and behavior toward this virus. Furthermore, this study shows that the increase of psychological burden seems to arise from a culmination of factors resulting from the pandemic, such as a subjective sense of uncertainty regarding medical care or financial stability, as well as some confounding factors, such as health status and neuroticism. The current situation is probably best described as a dilemma. Both the results of the pandemic, in terms of infection and illness, as well as countermeasures (social distancing), have negative consequences for our mental health. The results show that intensive work must be done on finding solutions – possibly through immunization *via* vaccination – as stressful moments caused by the pandemic contribute holistically to the psychological burden of the older population.

One limitation of this study is its cross-sectional design which restricts assumptions of causality. It cannot be assumed that the constructed model includes all influential factors modulating the relationship of COVID-19 experience in the social environment and psychological burden, which is depicted in the model fit. Furthermore, the construction of the variable psychological burden can be criticized, as it is based on several standalone items measuring adverse psychologic states rather than trialed psychological measures. This, however, is due to the largescale survey SHARE used as database of this study which assesses a multitude of variables to holistically depict the lives of persons pre- and postretirement in Europe. It is also important to stress that this study does not use before and after tests on psychological states. Therefore, burden changes only reflect the subjective perception of the respondents. Finally, the exclusion of persons who reported own experiences of COVID-19 may have led to an exclusion of relevant participants, as an infection in the environment is likely to coincide with own infection. However, as stated early on in this paper, these participants were excluded in order to avoid the contamination of the effect of experience of other with own experience.

Overall, this paper provides further evidence that illness experience in the environment, particularly the experience of the COVID-19 virus, adversely influences the psychological constitution and health. This study therefore reinforces prior findings provided by other international teams while adding information on the influences of other, previously overlooked factors pertaining to the reality of persons living in the time of COVID-19.

## Data Availability Statement

Publicly available datasets were analyzed in this study. This data can be found at: This paper uses data from SHARE waves 7 and 8 (DOIs: 10.6103/SHARE.w7.711, 10.6103/SHARE.w8ca.100), see Börsch-Supan et al. (2013) for methodological details and for data access http://www.share-project.org/.

## Ethics Statement

Ethical review and approval were not required for the study on human participants in accordance with the local legislation and institutional requirements. The patients/participants provided their written informed consent to participate in this study.

## Author Contributions

LR was the primary author of this manuscript. Analysis and writing were done in collaboration with TH. All authors contributed to the article and approved the submitted version.

## Funding

Under the terms of the Austria Open Access Publishing Framework Agreement, the St. Pölten University of Applied Sciences (Fachhochschule St. Pölten/FH St. Pölten) will cover Article Publishing Fees for eligible authors in any of the Frontiers journals. The SHARE data collection has been funded by the European Commission, DG RTD through FP5 (QLK6-CT-2001-00360), FP6 (SHARE-I3: RII-CT-2006-062193, COMPARE: CIT5-CT-2005-028857, and SHARELIFE: CIT4-CT-2006-028812), FP7 (SHARE-PREP: GA No. 211909, SHARE-LEAP: GA No. 227822, SHARE M4: GA No. 261982, and DASISH: GA No. 283646), and Horizon 2020 (SHARE-DEV3: GA No. 676536, SHARE-COHESION: GA No. 870628, SERISS: GA No. 654221, and SSHOC: GA No. 823782) and by the DG Employment, Social Affairs and Inclusion through VS 2015/0195, VS 2016/0135, VS 2018/0285, VS 2019/0332, and VS 2020/0313. Additional funding from the German Ministry of Education and Research, the Max Planck Society for the Advancement of Science, the United States National Institute on Aging (U01_AG09740-13S2, P01_AG005842, P01_AG08291, P30_AG12815, R21_AG025169, Y1-AG-4553-01, IAG_BSR06-11, OGHA_04–064, HHSN271201300071C, and RAG052527A) and from various national funding sources is gratefully acknowledged (see www.share-project.org).

## Conflict of Interest

The authors declare that the research was conducted in the absence of any commercial or financial relationships that could be construed as a potential conflict of interest.

## Publisher’s Note

All claims expressed in this article are solely those of the authors and do not necessarily represent those of their affiliated organizations, or those of the publisher, the editors and the reviewers. Any product that may be evaluated in this article, or claim that may be made by its manufacturer, is not guaranteed or endorsed by the publisher.

## Footnotes

^1^SHARE data collection is continuously ethically reviewed (see: http://www.share-project.org/fileadmin/pdf_documentation/SHARE_ethics_approvals.pdf). All studies using SHARE data uphold strict rules of participant protection.

^2^Persons were asked whether “anyone close to them” had lived through a COVID-19 infection or illness.

^3^7.293 respondents or 32% of the tested sample reported increased psychological burden since outbreak, of these 53% reported one, 33% two, and 14% three queried adverse states.
